# Extramedullary Plasmacytoma Involving the Heart: A Case Report and Focused Literature Review

**DOI:** 10.7759/cureus.7418

**Published:** 2020-03-25

**Authors:** Xuan Guan, Anum Jalil, Kishor Khanal, Baoqiong Liu, Akriti G Jain

**Affiliations:** 1 Internal Medicine, AdventHealth, Orlando, USA

**Keywords:** plasmacytoma, heart, echocardiography, computed tomography angiography, magnetic resonance imaging, chemotherapy, radiotherapy, positron emission tomography-computed tomography

## Abstract

Cardiac tumors are extremely rare. Here, we report an unusual case of cardiac plasmacytoma that occurred 11 years after complete remission of the original multiple myeloma (MM). The tumor primarily manifested as a solitary extramedullary plasmacytoma (SEP) with extensive infiltration into the heart and large vessels. There was no evidence of systemic involvement. The relapsing tumor assumed a unique immunophenotype from CD138+/CD38+/CD56- to CD138-/CD38+/CD56-. The patient responded to chemotherapy consisting of carfilzomib, cyclophosphamide, and dexamethasone. This case highlights the importance of multimodal imaging evaluation and tissue diagnosis for accurately characterizing this rare disorder.

## Introduction

Primary cardiac tumors are rare conditions. Only around 50 cases of cardiac plasmacytoma have been reported from 1977 to 2020 [1-3]. Thus, experience in disease diagnosis and management is limited. Here, we describe a case of relapsing cardiac plasmacytoma after chemotherapy and autologous stem cell transplantation presented as a solitary extramedullary plasmacytoma (SEP) invading the major intrathoracic cardiovascular structures.

## Case presentation

A 44-year-old female with no significant past medical history was admitted for atypical chest pain, described as a precordial non-exertional pressure sensation. The review of systems was largely negative. Vital signs were significant for mild tachycardia. Physical exam was significant for the presence of S3 and S4. The admission laboratory evaluation, as shown in Table 1, was only significant for chronic anemia, elevated N-terminal pro-brain natriuretic peptide (NT-BNP), and mildly elevated lactate dehydrogenase (LDH).

**Table 1:** Clinical laboratory results Ig: immunoglobulin; PE: protein electrophoresis; N-terminal pro-BNP: n-terminal pro-brain natriuretic peptide

Measure	Reference Range	Admission Lab	Interpretation
White-cell count (per μl)	4400-10,500	7670	-
Red-cell count (per μl)	3,750,000-5,000,000	4,000,000	-
Absolute neutrophil count (per μl)	1500-7500	3780	-
Absolute lymphocyte count (per μl)	1000-4800	3010	-
Platelet count (per μL)	139,000-361,000	297,000	-
Hemoglobin (g/dl)	11.4-14.7	10.1	Low
Hematocrit (%)	34.3-45.5	32.7	Low
Sodium (mmol/liter)	135-145	141	-
Potassium (mmol/liter)	3.5-5.0	3.9	-
Chloride (mmol/liter)	98-110	107	-
Calcium (mmol/liter)	8.5-10.5	8.9	-
Carbon dioxide (mmol/liter)	24-32	23	Low
Anion gap (mmol/liter)	5-15	11	-
Glucose (mmol/liter)	70-100	131	High
Blood urea nitrogen (mg/dl)	5-25	11	-
Creatinine (mg/dl)	0.6-1.2	1.02	-
Total protein (g/dl)	6.5-8.0	7.8	-
Albumin (g/dl)	3.2-5.5	4.3	-
Total bilirubin (mg/dl)	0.1-1.5	0.3	-
Alanine transferase (units/liter)	4-51	12	-
Aspartate transferase (units/liter)	5-46	15	-
Alkaline phosphatase (U/liter)	35-104	64	-
Troponin-T (ng/ml)	<0.03	<0.01	-
N-terminal pro-BNP (pg/ml)	0-450	726	High
Lactate dehydrogenase (U/liter)	60-200	239	High
Prothrombin time (sec)	11.5-14.9	14.1	-
International normalized ratio	0.8-1.2	1.13	-
IgG (mg/dl)	700-1600	1968	High
IgA (mg/dl)	70-400	118	-
IgM (mg/dl)	40-230	51	-
Beta 2 microglobulin (mg/l)	0.8-2.2	2.9	High
Serum protein electrophoresis			
PE total protein (g/dl)	6.5-8.0	7.4	-
PE albumin (%)	60.3-71.4	48.7	Low
PE alpha 1 (%)	1.4-2.9	5.4	High
PE alpha 2 (%)	7.2-11.3	9.3	-
PE beta (%)	8.1-12.7	11.9	-
PE Gamma (%)	8.7-16.0	24.7	High
PE absolute albumin (g/dl)	3.4-4.7	3.6	-
PE absolute alpha 1 (g/dl)	0.1-0.3	0.4	High
PE absolute alpha 2 (g/dl)	0.6-1.0	0.69	-
PE absolute beta (g/dl)	0.7-1.2	0.88	-
PE absolute gamma (g/dl)	0.6-1.6	1.83	High
M protein peak (g/dl)	0	0.94	High
Kappa light chain quantity	3.3-19.4	14.73	-
Lambda light chain quantity	5.7-26.3	14.82	-
Kappa/Lambda ratio	0.26-1.65	0.99	-

**Figure 1:**
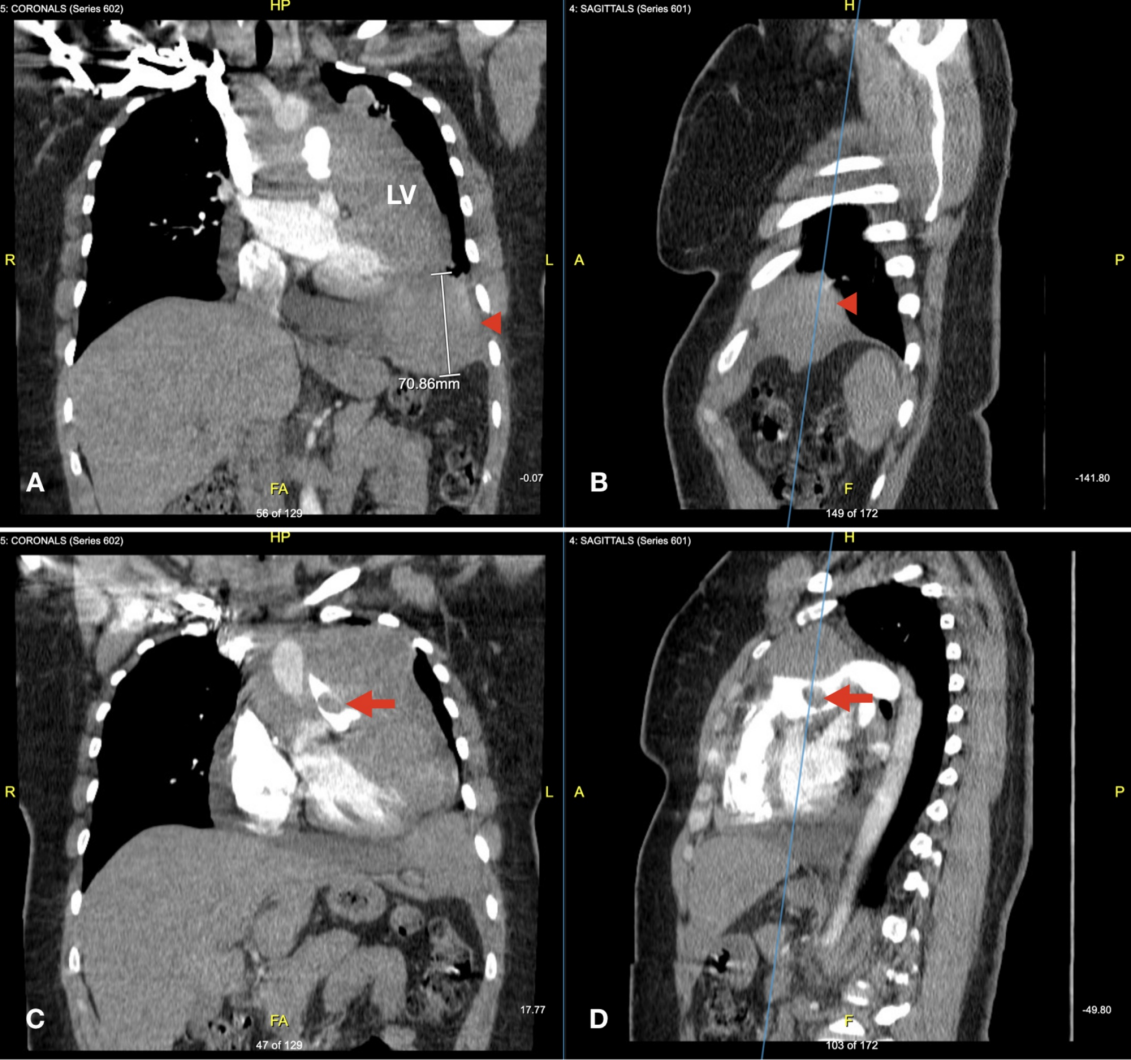
CT angiography coronal and sagittal reconstruction Coronal (A, C) and sagittal (B, D) reconstructions highlight the mass abutting the diaphragm (arrowhead) and infiltrating the left ventricle (LV) free wall (A & B), pulmonary artery involvement, and the intraluminal neoplastic thrombus (arrow) (C & D)

Computed tomography (CT) angiography (Figure 1) was negative for pulmonary embolism but incidentally revealed a moderate pericardial effusion with two mediastinal masses along the left cardiophrenic angle and within the superior pericardial recess. Additional infiltration was found along the left aspect of the pericardium, left ventricular myocardium, left hemithorax pleura, and the lateral left wall of the main pulmonary artery with an endoluminal component partially obstructing the lumen.

**Figure 2:**
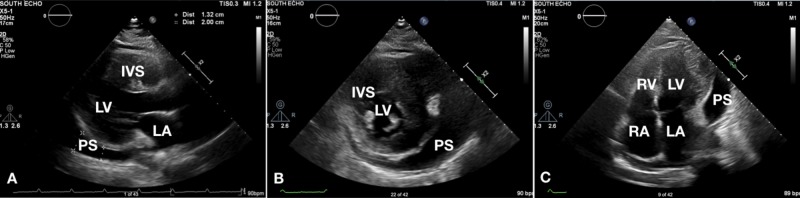
Representative echocardiographic images Parasternal long (A), parasternal short (B), and apical four-chamber views demonstrate pericardial effusion, thickened left ventricle wall, and interventricular septum LA: left atrium, LV: left ventricle, RA: right atrium, RV: right ventricle, IVS: interventricular septum, PS: pericardial effusion

Echocardiogram (Figure 2) confirmed severe left ventricle (LV) hypertrophy, mostly affecting the lateral wall, apex, and interventricular septum with impaired LV diastolic filling.

**Figure 3:**
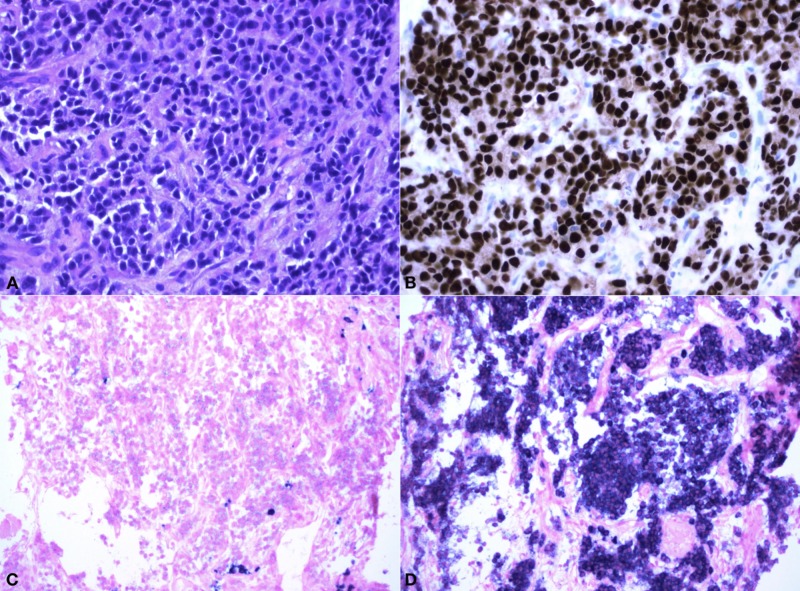
Representative pathology micrographs of core needle biopsy (40x) (A) Hematoxylin & Eosin (H&E) staining; (B) MUM1 immunochemistry staining showing positive tumor cells; (C) *in-situ* hybridization showing tumor cells are negative for Kappa light chain; (D) in-situ hybridization showing tumor cells are positive for Lambda light chain

In order to establish a tissue diagnosis, a CT-guided biopsy was performed by interventional radiology. Pathology revealed the tumor was positive for multiple myeloma oncogene 1 (MUM1) and lambda light chain, consistent with plasma cell neoplasm (Figure 3).

**Figure 4:**
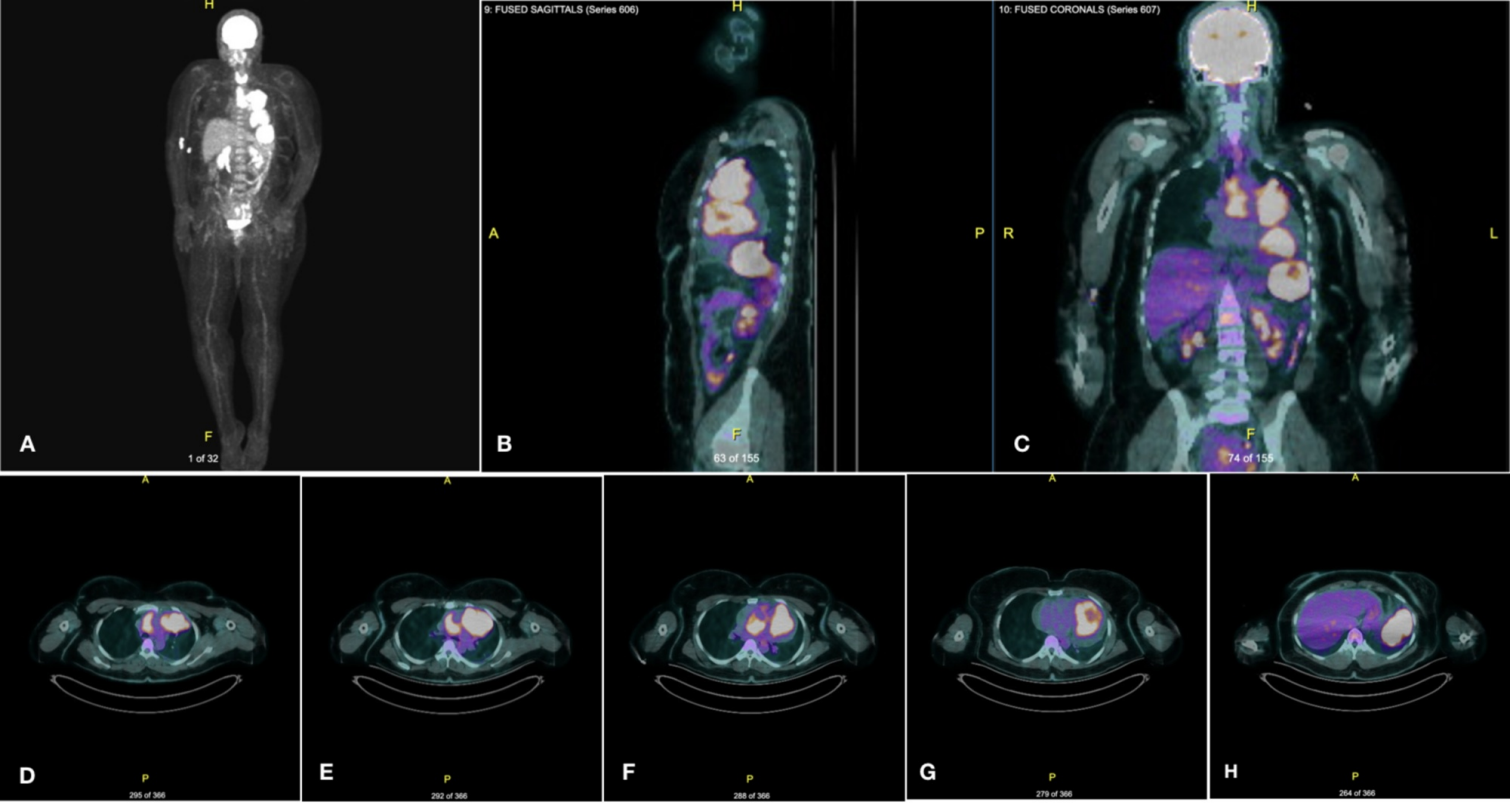
Representative PET/CT scan Maximal intensity projection (MIP) display (A), fused sagittal view (B), fused coronal view (C), and fused horizontal tomographic renditions (D-H) demonstrate intrathoracic neoplastic burden, highlighting the tumor’s close proximity and direct invasion of cardiac and vascular structures PET/CT: positron emission tomography-computed tomography

Flow cytometry demonstrated cytoplasmic lambda-restricted monoclonal plasma cells that were CD138-, CD38+, CD19-, CD20-, CD56-, CD117-, and CD45-. Serum immunoglobulin quantification (Table 1) revealed elevated immunoglobulin G (IgG) and beta2 microglobulin. Serum protein electrophoresis and immunofixation were significant for monoclonal M protein and elevated IgG alpha 1 (Table 1). In the myeloma workup, the complete bone survey was negative for lytic bone lesions while bone marrow biopsy and flow cytometry revealed no monoclonal plasma cells. Furthermore, positron emission tomography/computed tomography (PET/CT) (Figure 4) confirmed the intrathoracic hypermetabolic lesion without metastasis or bony involvement.

Based on the above findings, SEP was diagnosed due to the absence of plasma cell monoclonality or myeloma defining features. Immunoglobulin-producing plasma cell tumors are known to cause amyloid deposition in various organs including the heart [1,4]. In order to rule out cardiac amyloidosis, a cardiac MRI was ordered and demonstrated the mass infiltrated and replaced most of the anterior, anteroseptal, and anterolateral wall of the LV, resulting in myocardial stiffening with limited radial contraction at the level of the infiltration. Moreover, there was encasement, extrinsic compression, and moderate narrowing of the pulmonary trunk, right pulmonary artery, aortic arch, and right brachiocephalic artery. The pulmonary trunk was directly invaded by a 1.98 cm mobile endoluminal thrombus.

Upon reviewing the patient’s previous medical record, it was found that the patient was admitted 11 years ago for a two-week history of epigastric discomfort, bilateral lower extremity edema, and a 40-pound unintentional weight loss. CT chest demonstrated an approximately 7 cm mass abutting the left ventricle, laterally extending to the left lung, and multiple mediastinal lymph nodes and nodularity throughout the pericardium. Fine needle aspiration (FNA) revealed a monotonous plasma cell population that was CD138+ with restricted monoclonal lambda light chain expression. Cardiothoracic surgery was consulted for an anterior mediastinotomy. Direct cardiac structures invasion was visualized intraoperatively. Pathology confirmed a neoplastic infiltration of the epicardium and pericardium. A bone survey revealed several lucent lesions in the frontal calvarium. Serum immunoglobulin G (IgG) was elevated at 2,090 ng/dL. Despite negative bone marrow biopsy, the patient was diagnosed with MM (biopsy confirmed extramedullary plasmacytoma and bony lesions). She was treated with Revlimid and zoledronic acid for a short period of time. Despite the treatment, the mediastinal mass continued to grow. Hence, the regimen was subsequently switched to cyclophosphamide, bortezomib, and dexamethasone for a total of six cycles. Follow-up PET/CT demonstrated significant improvement of the mediastinal mass and osseous lesions. Then, autologous stem cell transplantation was performed following intravenous melphalan. The patient remained in remission for 11 years, evident by multiple negative surveillance CT scans and bone marrow biopsies, before this relapse.

The possibility of radiation therapy was evaluated after the diagnosis of relapsing cardiac SEP. The risk of cardiotoxicity was deemed too high. Hence, the patient was started on chemotherapy, entailing carfilzomib, cyclophosphamide, and dexamethasone. She was discharged after one cycle and has been following with oncology as an outpatient for continued treatment. The patient responded favorably, as demonstrated by the shrinking mass and normalized serum IgG four months post-discharge. She remained largely asymptomatic after seven months.

## Discussion

The incidence of primary cardiac tumors was reported to be less than 0.1% in a series of 12,000 autopsies. Metastatic tumors are about 20 times more common than primary cardiac tumors [5].

Plasmacytic neoplasms encompass a wide spectrum of disorders, ranging from systemic multiple myeloma, Waldenstrom's macroglobulinemia, to localized plasmacytoma. Extramedullary plasmacytoma (EP) can either be a concurrent presentation of MM (7%) or develops later after the diagnosis of MM (6%). SEP, a localized plasma dyscrasia arising outside of bone marrow, accounts for only 3% of all plasma cell disorders. Diagnosis of SEP requires excluding the features of MM, including (1) free of anemia (hemoglobin < 10), hypercalcemia, or renal impairment; (2) PET/CT scan demonstrating no lytic bone lesions, and (3) absence of clonal plasma cells from bone marrow biopsy. EP and SEP most often involve the upper respiratory tract, soft tissue, and gastrointestinal tract. Cardiovascular involvement is exceedingly rare. A review of the literature from 1977-2020 resulted in approximately 50 cases, most of which have been summarized by Coakley et al., Keung et al., and Vigo et al. [1-3]. Among these pathology-confirmed cases, two-thirds had a previous history of plasma cell disorders, such as MM, monoclonal gammopathy of undetermined significance (MGUS), or plasmacytoma. Primary cardiac SEP only accounts for approximately 4%. Interestingly, cardiac plasmacytoma demonstrated a predilection of the right atrium (42%) while pericardial effusion is the most common presentation (87.8%). On the other hand, direct myocardium infiltration, as in our case, is relatively rare (9%). To the best of our knowledge, extensive neoplastic infiltration of cardiovascular structures encompassing the myocardium, great vessels, epicardium, and pericardium in a similar fashion to the current case has never been reported. 

Another feature of the current case is its unique immunophenotype. Tumor cells’ surface markers switched from CD138+/CD38+/CD56- to CD138-/CD38+/CD56- upon relapse. CD56 is a marker of abnormal plasma cells. Seventy-five percent (75%) of normal plasma cells are CD56 negative and 70% of MM cells are CD56 positive [6]. In our cases, both primary and recurrent tumor cells were CD56 negative. It has been previously reported that negative CD56 is more common in plasma cell leukemia, an aggressive extramedullary plasma cell neoplasm. Several studies reported a reduced CD56 expression in EMPs as compared to MM or solitary plasmacytoma of bone [7-9]. It is hypothesized that, as a surface adhesion molecule, CD56 plays a role in the bone marrow localization of tumor cells and its down-regulation may account for its extramedullary localization [10]. CD138 is a surface marker of both normal and neoplastic plasma cells. Reduced CD138 expression is reported in progressive/relapse MM. Patients with low CD138 expression had worse outcomes. MM cells with low CD138 possess an immature “stem-cell-like'' molecule profile and demonstrate increased resistance to lenalidomide [11]. In vitro experiments have demonstrated that conditions such as bone marrow stromal cell interaction and hypoxia may contribute to CD138 down-regulation, which is associated with resistance [12-13]. 

Radiation therapy (RT) is widely regarded as the first-line treatment for SEP. Surgical resection could be considered in the case of localized tumors that are amenable to excision. Surgery can be combined with RT if complete resection is not possible. The role of chemotherapy is controversial and usually has no benefit in inducing or maintaining relapse. It might slow down the rate of progression of plasmacytoma to multiple myeloma, but it has no benefit in reducing the rate of conversion [14-16]. In our case, due to the location of the tumor and the potential risk of cardiotoxicity, a decision was made against RT and the patient was managed with chemotherapy alone. The concern of radiation cardiotoxicity was also shared by Vrettou et al., who reported a patient with recurrent plasmacytoma involving the heart receiving six cycles of bortezomib, cyclophosphamide, and dexamethasone to “debulk” prior to RT [17]. However, RT is still the treatment of choice in the majority of reported cases. Second treatment modalities, such as surgical excision, systemic/local intrapericardial chemotherapy, or high-dose steroid, are often included as adjunct therapy [2,4,18-19]. Single modality treatment is relatively rare and the outcomes were generally poorer [1,20]. Vigo et al. reviewed 11 cases and concluded that RT combined with systemic therapy resulted in better outcomes [3]. However, no statistical analysis was conducted to support this view. Admittedly, appraisal of the therapeutic outcome can be difficult due to the scarcity of this disorder.

## Conclusions

Despite the low incidence of cardiac plasmacytoma, it is important for clinicians to consider the possibility of the direct neoplastic involvement of cardiovascular structures, especially in patients with a previous history of plasma cell disorders presenting with related symptoms. Functional and anatomical assessment via various imaging modalities, together with a tissue diagnosis, is fundamental in determining the treatment options. In our case, the patient responded favorably to chemotherapy. However, the literature suggests RT-based multimodal therapy may be associated with superior outcomes. Adequately powered future studies are warranted to evaluate the efficacy of various treatment modalities in managing cardiac SEP.
